# Scalp Angiosarcoma Remission with Bevacizumab and Radiotherapy without Surgery: A Case Report and Review of the Literature

**DOI:** 10.1155/2011/160369

**Published:** 2011-05-02

**Authors:** Jocelyn T. De Yao, Danyu Sun, Angela T. Powell, Esther H. Rehmus

**Affiliations:** ^1^Department of Medicine, Akron General Medical Center, 400 Wabash Avenue, Akron, OH 44307, USA; ^2^Department of Medicine, Northeastern Ohio Universities, College of Medicine and Pharmacy, Rootstown, OH 44272, USA; ^3^Department of Pathology, Akron General Medical Center, 400 Wabash Avenue, Akron, OH 44307, USA

## Abstract

Angiosarcoma (AS) is a rare and aggressive vascular neoplasm with very poor prognosis. Patients with extensive cutaneous AS who are not surgical candidates have very limited options since there is no standard treatment. Treatment options include radiation, chemotherapy, and angiogenesis inhibitor with varying success rates. Here, we report a case an 88 year old patient with extensive scalp angiosarcoma having biopsy proven remission with bevacizumab and radiotherapy without undergoing surgery.

## 1. Introduction

Angiosarcoma (AS) is a rare and aggressive vascular neoplasm comprising less than 2% of all soft tissue sarcomas [1–3]. Angiosarcomas comprise approximately 15% of all head and neck sarcomas [4–6] and less than 1% of all malignancies in the region [6, 7]. Head and neck ASs are frequently seen in the scalp [1–3, 6–15] and are common in the elderly population with the mean age greater than 60 years. AS also has a slight predilection for Caucasian patients and the male gender [1, 2, 7–10, 12–14]. Prognosis is poor since AS is very aggressive with disease-free survival of less than 50% at five years [1, 4, 5, 8–14] due to its high propensity for local recurrence [4, 5, 8, 11]. However, some studies report survival of 10–20% after five years [1, 5, 8–11], which may indicate lesions that are located in the scalp and face have a lower survival rate and higher rate of recurrence [10]. The most important prognostic factors include tumor size and ability to achieve negative margins with tumor grade also being considered to be a prognostic factor for recurrence [5, 12] and overall survival [11]. Patients with facial or scalp AS of less than 5 cm in size tend to do better than those with tumor size greater than 5 cm [6, 10]. Patients with cutaneous AS who are not surgical candidates due to multiple comorbid conditions have limited treatment options. Due to its rapid progression, achieving local control and prevention of metastasis in AS has been a challenge. Few reports have described successful treatment of AS using adjuvant chemotherapy with radiotherapy [16]. Different treatment modalities used in cutaneous angiosarcoma are depicted in Table 1. Here, we report a successful response of AS to bevacizumab with concurrent radiation therapy in a patient not eligible for surgical intervention.

## 2. Case Report

**Figure 1:**
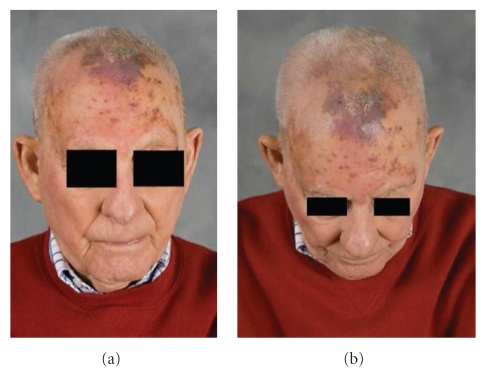


An 88-year-old Caucasian male with a 37-pack-year history of smoking noticed purplish lumps on his scalp with scaling and erythema for two months prior to presentation. He had a complicated past medical history of Stage II prostate cancer treated with radiation treatments, abdominal aortic aneurysm repair, macular degeneration treated with photodynamic therapy, torn retina, inguinal hernia repair, and four-vessel coronary artery bypass graft. The patient noted the violaceous papules increasing in size and nodularity and extending down to the level of the eyebrows bilaterally. He initially thought the mass was a bruise, but the increase in size prompted him to consult a physician. On examination, the patient had multiple irregularly shaped lesions, largest of which was a 4 cm × 7 cm on the frontal scalp; these lesions were violaceous papules with scaling and erythema as shown in Figure 1. A PET scan showed uptake only on the scalp adjacent to the frontal bone near midline (Stage IIA—T2a N0 M0).

## 3. Histology

**Figure 2:**
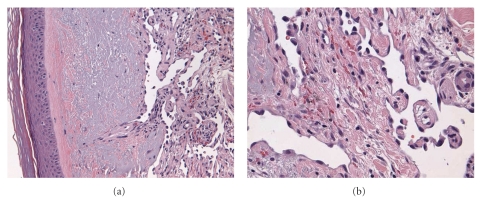


Biopsy of the lesion showed irregular channels and ectatic vascular spaces lined by plump hyperchromatic endothelial cells. The histology results were most compatible with moderately differentiated angiosarcoma, as shown in Figure 2, in low magnification (L) and high magnification (R).

## 4. Treatment and Followup

**Figure 3:**
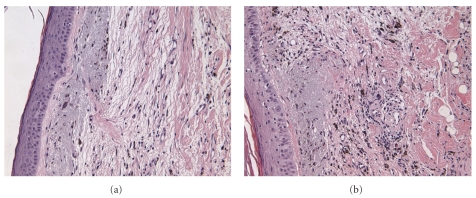


Due to the extent of the disease and his frail state, the patient was a poor surgical candidate; resection, if performed, would have necessitated the removal of his entire scalp, from ear to ear. The patient was started on one pre-irradiation cycle of bevacizumab, which was continued through his course of radiation therapy. Bevacizumab was given 5 mg/kg IV (with calculated dose of 350 mg) every two weeks while on radiation therapy, total of four treatments. His tumor total radiation treatment dose was 6000 rad, in 30 fractions, over 46 days. The patient tolerated the treatment well with skin changes characterized only by erythema and desquamation with residual crusting of the skin. Marked clinical improvement was noted. Side effects also included slight swelling of the eyes with yellowish discharge and pruritus from the radiation treatment. No hypertension or proteinuria was noted. A repeat biopsy performed six months after treatment showed fibrosing granulation tissue, fibrosis, hemosiderin deposition, and dilated vessels not reminiscent of the pattern seen in the original angiosarcoma as shown in Figure 3.

Seven months after the completion of bevacizumab and radiation therapy, the patient had a biopsy-proven local recurrence. He was started immediately on an increased dose of bevacizumab 10 mg/kg IV every two weeks, which he tolerated well for three months for a total of 6 doses. He then had a minor stroke, and bevacizumab was discontinued. Weekly paclitaxel was initiated at 80 mg/m^2^ and continued for 15 treatments with stable disease. Progression occurred and treatment was changed to pegylated doxorubicin 40 mg/m^2^ monthly × 2 treatments without benefit. He expired 23 months after initial presentation due to disease.

## 5. Discussion

Angiosarcoma usually presents as a purple macular ulcerated lesion that is often mistaken for a bruise. Cutaneous angiosarcoma may be categorized as being idiopathic AS of the face and scalp, primary AS of the breast, postradiation AS, or AS arising from chronic lymphedema of the extremity [9, 10, 23]. Proposed risk factors for developing cutaneous AS include exposure to vinyl chloride, insecticides, thorium dioxide, and hormones such as anabolic steroids and synthetic estrogen [5, 12]. It is also postulated that sun-damaged skin may be a risk factor due to its predilection for Caucasians; however, there is no direct evidence to support this theory [1, 23]. A combination of surgery and radiotherapy offers the best survival and is the most common treatment modality used for this very aggressive tumor [9, 12]. Surgery remains the first option for treating patients with angiosarcoma of the scalp, but due to its rapid progression, patients may present with disseminated or extensive local disease which make surgery inappropriate [1, 8–15]. Postoperative radiotherapy is employed routinely as it improves survival [5, 7, 8], although there is still an extremely high rate of recurrence and local failure [12]. The role of chemotherapy, however, is not well established and has not been proven to increase survival [12]. 

As a result of the above-mentioned challenges, treatment options are limited, especially since AS is often found in elderly patients who may have many comorbidities and are more prone to adverse treatment reactions. Prospective studies with regard to adjuvant chemotherapy and other treatment combinations are also limited due to the rarity of the disease so there are no specific guidelines with regards to treatment. Radiation therapy dose response has been suggested wherein those receiving greater than 5000 rad have better local control, though larger case numbers are required to validate initially reported successes [5]. There are case reports of remission with radiotherapy alone; these cases tend to be well-differentiated and exophytic forms of AS [17]. 

The addition of another therapy to radiation therapy has seen some support over the years; different cases showed AS responding to radiation when biological, angiomodulation, and/or tubulin-affinic substances are given in conjunction with radiation therapy [5, 16, 18–20, 23–26]. Nevertheless, the overwhelming opinion about chemotherapy is that its utility remains largely undefined [5]. Major responses have also been reported with paclitaxel, but side effects are dose limiting [24, 25]. A retrospective study done on 32 advanced angiosarcoma patients by the soft tissue and bone sarcoma EORTC group has 62% response rate in advanced angiosarcomas with paclitaxel, with 6 out of 8 scalp and face angiosarcomas responding [18]. There are 6 scalp angiosarcoma patients in the EORTC study, with 3 previously treated with radiation therapy (2 with partial response and 1 with progression of disease) and 1 previously treated with doxorubicin (no change with paclitaxel). However, there has been a complete response for 42 months on one patient with scalp angiosarcoma without previous therapy and no previous surgery. Another taxane drug, docetaxel, has been used for cutaneous AS; a retrospective study of nine patients in Japan reported a major response in six patients with reduced neutropenia and peripheral neuropathy compared to paclitaxel [19]. Published case reports have cited a partial response without surgery [26] and a sustained complete response after surgery in a patient four years later [20] following the addition of liposomal doxorubicin [20, 21, 26]. Eiling et al. reported a case of confirmed histological tumor regression of a nonresectable radioresistant scalp angiosarcoma and cervical lymph node metastasis treated with pegylated liposomal doxorubicin [21]. Complete remission with liposomal daunorubicin with radiotherapy in unresectable AS has also been published [22].

Although our patient had a moderately differentiated AS, the tumor location, the extent of disease, the patient's age, and his comorbidities all pointed to a very poor prognosis. Due to the extent of our patient's disease, it would have been difficult to achieve a tumor-free margin surgically without any disfiguring effects, and radiotherapy alone was initially thought to be insufficient. It is difficult to assess whether our patient's response is due to radiotherapy alone similar to the case reported by Gkalpakiotis et al. [17] or if bevacizumab improved our patient's response since vascular endothelial growth factor was overexpressed in 80% of angiosarcoma cases [27]. To our knowledge, this case is the only one to report remission of AS after treatment with bevacizumab and radiotherapy without surgical intervention. In two case reports from the Duke University, 5 mg/kg of bevacizumab was used in one patient and 10 mg/kg for the other in conjunction with radiotherapy as postsurgical treatment [16]. Our patient was given four treatments of 5 mg/kg of bevacizumab with radiation therapy as a primary treatment without surgical resection and achieved complete clinical response with a negative pathology on biopsy remission. Due to the lack of resection, we cannot report a complete pathologic response. A second course involving a higher dose of bevacizumab for his recurrence led to clinical response after three months but had to be discontinued when patient had a stroke, a known risk of bevacizumab though a meta-analysis done based on 5 randomized controlled trials among 2,288 patients receiving bevacizumab showed no statistical difference of stroke incidence compared to control [28]. 

With AS being very aggressive and having a very low five-year survival rate, therapy with bevacizumab in conjunction with radiation therapy or as a primary therapy may be one option to improve survival outcomes especially in patients who are not surgical candidates. Currently, there are two phase II clinical trials studying the use of bevacizumab for AS which were not available during our patient's initial diagnosis. One is an open-label multicenter phase II trial assessing the median disease-free progression of AS with the treatment of bevacizumab alone [29]. The other is the study of adding bevacizumab to the combined chemotherapy of gemcitabine and docetaxel in the treatment of different types of sarcoma including AS [30]. Both trials are currently recruiting patients. A prospective clinical trial regarding neoadjuvant therapy with bevacizumab possibly also including chemotherapy would be an interesting approach. In the absence of specific treatment guidelines for AS, the addition of bevacizumab to radiation may be one option to offer hope to patients who are deemed nonsurgical candidates.

## Figures and Tables

**Table 1:** Different treatments used in cutaneous angiosarcoma.

Reference	Type of literature	Angiosarcoma type	Treatment	Outcome
Koontz et al. 2008 [16]	Case reports	(1) Nasal area	(1) Bevacizumab + XRT + SX	(1) CR, 26 mos response duration
		(2) Nasal area	(2) Bevacizumab + XRT + SX	(2) CR, 8.5 mos response duration

Gkalpakiotis et al. 2008 [17]	Case report	Well-differentiated exophytic-face	XRT alone Phase 1: 45 Gy Phase 2: 20 Gy	5 yrs remission

Schlemmer et al. 2008 [18]	Retrospective study	32 patients	Paclitaxel	5/8 PR (mean 5.8 mos)
		8 scalp and facial	± Sx	1/8 CR (42 mos)
		Rest other sites	± XRT	1/8 PD (3 mos)
			± chemotherapy	1/8 NC (2 mos)

Nagano et al. 2007 [19]	Retrospective study 9 patients	Cutaneous AS	Docetaxel with or without other previous treatment (XRT, SX)	6/9: major response (2 CR, 4 PR)
				2CR: 2 XRT without SX
				4 PR: 1 XRT + SX, 1 XRT without SX, 1 SX without XRT, 1 without SX nor XRT

Holloway et al. 2005 [20]	Case report	Cutaneous AS (scalp)	Liposomal doxorubicin + XRT	4 years response duration

Eiling et al. 2002 [21]	Case report	Cutaneous AS (scalp)	Liposomal doxorubicin + XRT	CR with cervical lymph node metastasis disappearance
				4 mos response duration

Lankester et al. 1999 [22]	Case report	Cutaneous AS (face and scalp)	Liposomal daunorubicin + XRT	15 mos CR

CR: complete response, PR: partial response, NC: no change, PD: progression of disease, XRT: radiotherapy, SX: surgery.
